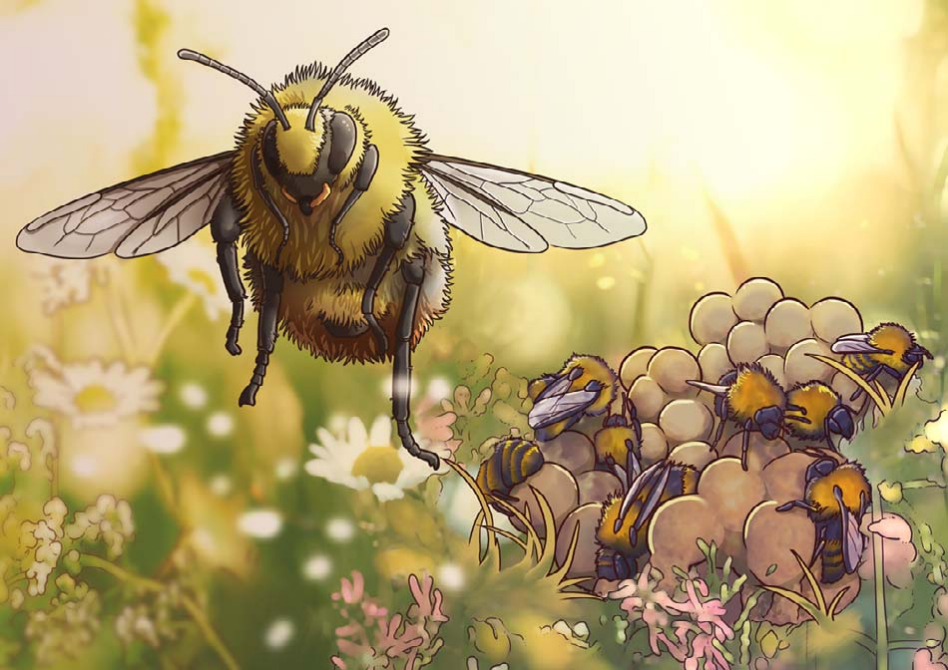# New queens can’t run the bee world without a healthy diet

**DOI:** 10.1093/conphys/coaa007

**Published:** 2020-04-28

**Authors:** Rachael M Heuer

**Affiliations:** Rosenstiel School of Marine and Atmospheric Science, University of Miami, 4600 Rickenbacker Causeway, Miami, FL 33149, USA

It is no secret that bees play an important role in human lives, pollinating wild plants as well as those that humans consume, like blueberries and tomatoes. Unfortunately, bees face a number of threats that have led to massive declines in their populations, putting us at risk of losing many of the important services that bees provide. One fundamental part of maintaining bee health is making sure bees have access to a healthy diet. This is especially important for the new queen, since she is responsible for producing offspring in the colony. A new study by Hollis Woodard and her team demonstrates that a poor diet early in a queen bee’s life can have long-lasting negative impacts on survival.

The bumble bee queen life cycle is typically broken into three phases. After new queens hatch, they spend about 1 week consuming and storing nutrients to prepare for the winter before mating. In the second phase, the queen enters an inactive state to cope with harsh temperatures during the winter. In the spring, the queen will emerge and start a new bee hive. Woodard’s team examined how the bumble bee queen’s diet during the short, first phase of life can influence fitness in all stages.

The team first designed an experiment where they could manipulate the diet of queen bees. In an ideal scenario, queens would have access to both pollen and nectar. Pollen provides protein and lipids (fats), while nectar provides carbohydrates (sugars). Among the diets that Woodard designed included a control diet with normal levels of both pollen and nectar, a diet lacking pollen and a diet lacking nectar. Newly hatched queens were then fed one of these diets for the following 11 days.

Woodard and team measured weight and survival over the short 11-day period. Queens on the ‘no-nectar diet’ were much lighter than queens with access to both nectar and pollen (the control diet). In addition, queens on the control diet were more likely to survive than queens without access to pollen.

The team then wanted to see if the diets had lasting effects across other life stages. To test this idea, they simulated inactive winter conditions (i.e. temperatures of a typical refrigerator) by chilling the bees for 2 months. Queens in the no-pollen and no-nectar diets experienced much lower survival than control queens during this period. Surprisingly, queens that were nectar-deprived before winter continued to show reduced survival after being ‘awakened’ and fed a healthy diet.

What do these findings mean for the health of bumble bees? Woodard and her team point out that their observations of immediate and lasting negative effects of poor diet on queen health and survival means that access to quality nutritional resources during the narrow time window at the beginning of a queen’s life is extremely important. This study not only provides key insight into queen nutritional physiology but also can be used by conservation managers. Managing high-quality floral food sources close to bee habitat is one more strategy in the toolkit to try and prevent further declines in bumble bee populations.

Illustration by Erin Walsh; Email: ewalsh.sci@gmail.com